# Omicron infection-associated T- and B-cell immunity in antigen-naive and triple-COVID-19-vaccinated individuals

**DOI:** 10.3389/fimmu.2023.1166589

**Published:** 2023-05-05

**Authors:** Joana Barros-Martins, Swantje I. Hammerschmidt, Gema Morillas Ramos, Anne Cossmann, Laura Hetzel, Ivan Odak, Miriam Köhler, Metodi V. Stankov, Christiane Ritter, Michaela Friedrichsen, Inga Ravens, Anja Schimrock, Jasmin Ristenpart, Anika Janssen, Stefanie Willenzon, Günter Bernhardt, Ralf Lichtinghagen, Berislav Bošnjak, Georg M. N. Behrens, Reinhold Förster

**Affiliations:** ^1^ Institute of Immunology, Hannover Medical School, Hannover, Germany; ^2^ Department for Rheumatology and Immunology, Hannover Medical School, Hannover, Germany; ^3^ Department of Clinical Chemistry, Hannover Medical School, Hannover, Germany; ^4^ German Center for Infection Research (DZIF), Partner Site Hannover-Braunschweig, Hannover, Germany; ^5^ Centre for Individualized Infection Medicine (CiiM), Hannover Medical School, Hannover, Germany; ^6^ Cluster of Excellence RESIST (EXC 2155), Hannover Medical School, Hannover, Germany

**Keywords:** SARS-CoV-2, COVID-19, Omicron variants, Omicron infection, breakthrough infection, heterologous vaccination

## Abstract

Since early 2022, various Omicron variants have dominated the SARS-CoV-2 pandemic in most countries. All Omicron variants are B-cell immune escape variants, and antibodies induced by first-generation COVID-19 vaccines or by infection with earlier SARS-CoV-2 variants largely fail to protect individuals from Omicron infection. In the present study, we investigated the effect of Omicron infections in triple-vaccinated and in antigen-naive individuals. We show that Omicron breakthrough infections occurring 2–3.5 months after the third vaccination restore B-cell and T-cell immune responses to levels similar to or higher than those measured 14 days after the third vaccination, including the induction of Omicron-neutralizing antibodies. Antibody responses in breakthrough infection derived mostly from cross-reacting B cells, initially induced by vaccination, whereas Omicron infections in antigen-naive individuals primarily generated B cells binding to the Omicron but not the Wuhan spike protein. Although antigen-naive individuals mounted considerable T-cell responses after infection, B-cell responses were low, and neutralizing antibodies were frequently below the limit of detection. In summary, the detection of Omicron-associated B-cell responses in primed and in antigen-naive individuals supports the application of Omicron-adapted COVID-19 vaccines, but calls into question their suitability if they also contain/encode antigens of the original Wuhan virus.

## Introduction

Coronavirus disease 2019 (COVID-19) is caused by infection with a novel coronavirus known as severe acute respiratory syndrome coronavirus type 2 (SARS-CoV-2). Despite a successful vaccination campaign starting at the end of 2020, infections with SARS-CoV-2 reached unprecedented levels in many countries in the first months of 2022 due to the spread of several, highly infectious, new Omicron sublineages of SARS-CoV-2. Compared with the spike protein of the Wuhan-Hu-1 strain, the spike proteins of the different Omicron variant sublineages contain 37 (or more) different amino-acids (1). These differences give all Omicron sublineages the ability to evade the immune system, as prior vaccination or infection with other variants provides little protection against Omicron infections. However, fully vaccinated individuals are still largely protected from severe disease manifestation requiring hospitalization or even treatment in an intensive care unit.

The majority of currently licensed COVID-19 vaccines are based on the spike protein derived from the original Wuhan-Hu-1 virus or have been developed by inactivation of viral particles of this strain. Based on the analysis of antibodies and virus-specific T cells, as well as real real-world data, health agencies such as the Food and Drug Administration of the USA, the European Medicines Agency of the European Union, and Germany’s Paul Ehrlich Institute recommend three vaccine injections in adults for best protection against COVID-19. For elderly and/or immune-compromised patients, as many as four vaccine shots are deemed necessary.

All vaccines were originally licensed solely for homologous application, i.e., two or more injections of the same vaccine. However, in several countries, vaccination with AstraZeneca’s Vaxzevria (ChAd) was halted in spring 2021 due to vaccine-induced thrombotic thrombocytopenia (2). Therefore, many countries recommended that vaccinees who received a first dose of ChAd should subsequently receive an RNA-based vaccine from BionTech/Pfizer (Comirnaty; BNT) or Moderna (spikevax). Such immunization protocols, now known as heterologous immunization, are safe and frequently induce immune responses stronger than those observed after homologous immunization (3–6).

We recently reported on immune responses in a cohort of healthcare professionals after homologous and heterologous immunization with ChAd/ChAd, ChAd/BNT, or BNT/BNT (3, 7) and described how a third immunization with BNT affected their immune response (7). As immunity wanes over time, further booster vaccines, some of which have been adapted to Omicron sublineages, are now being applied to reduce the risk of severe COVID-19. However, it is still not entirely clear to what extent previous COVID-19 vaccinations or natural infections with the various SARS-CoV-2 variants imprint on the immune response and whether they interfere with the generation of Omicron-specific B and T cells. In the present study, we assess the effect of a SARS-CoV-2 breakthrough infection, with the Omicron lineage, in ChAd/BNT/BNT vaccinees 17–19 weeks after the third vaccine administration. Breakthrough infections were accompanied by mild clinical symptoms or were clinically unapparent. These infections induced a pronounced immune response, with antibody titers and spike-specific T-cell frequencies being restored to, or even surpassing, levels observed 2 weeks after the third vaccination. In contrast, non-vaccinated individuals infected with an Omicron variant mounted only CD4-mediated immune responses, primarily against epitopes of the viral membrane (M), nucleocapsid (N), or envelope (E) protein, and frequently failed to produce neutralizing antibodies.

## Materials and methods

Parts of this section have been reproduced or adapted from our previous work (3, 7), which is distributed in accordance with the terms of the Creative Commons Attribution (CC BY 4.0) license, which permits others to distribute, remix, adapt, and build upon this work, for commercial use, provided the original work is properly cited. See: http://creativecommons.org/licenses/by/4.0/.

### Participants

**Figure 1:**
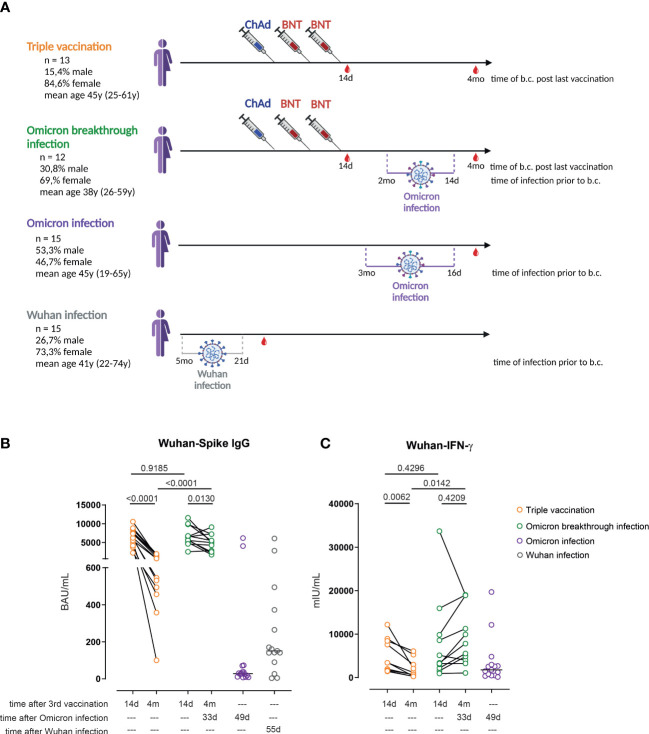
Omicron breakthrough infection in ChAd/BNT/BNT vaccinees restores anti-spike IgG antibodies and T-cell responses. **(A)** Participant recruitment scheme; “time post infection” reflects the mean time (and range) between infection and blood collection. **(B)** Anti-S1 IgG levels measured by enzyme-linked immunoassay (ELISA) in the plasma of ChAd/BNT/BNT vaccinees without Omicron breakthrough infection (triple vaccination, orange), ChAd/BNT/BNT vaccinees after Omicron breakthrough infection (Omicron breakthrough infection, green), non-vaccinated individuals after Omicron infection (Omicron infection, purple), and non-vaccinated individuals after Wuhan infection (Wuhan infection, gray). **(C)** IFN-γ concentration in whole blood supernatants after stimulation with the SARS-CoV-2 S1 domain (ancestral strain) for 20–24 h measured by IGRA. **(B, C)** Paired *t*-test (within groups) or two-way ANOVA followed by Sidak’s multiple comparison test (between groups); lines represent group median. **(A)** created with BioRender.com. ChAd, AstraZeneca’s Vaxzevria vaccine; BNT, BionTech/Pfizer’s Comirnaty vaccine.

Participants for this analysis were from the COVID-19 Contact (CoCo) Study (German Clinical Trial Registry, DRKS00021152), an ongoing, prospective observational study monitoring anti-SARS-CoV-2 IgG and immune responses in healthcare professionals (HCPs) at Hannover Medical School and individuals in potential contact with SARS-CoV-2 (8, 9). An amendment in December 2020 allowed us to study immune responses after COVID-19 vaccination. We followed the study cohort described previously (3, 7) after heterologous ChAd/BNT/BNT vaccination. Data, including responses to questionnaires and the results of laboratory assessment, were recorded in Excel 2016. Demographics (sex and age) are depicted in **Figure 1A**. After blood collection, we separated the plasma from EDTA or lithium heparin blood (S-Monovette, Sarstedt) and stored it at –80°C until use. We used whole blood or peripheral blood mononuclear cells (PBMCs) isolated from whole-blood samples using Ficoll gradient centrifugation for stimulation with SARS-CoV-2 peptide pools. The number of biologically independent samples analyzed in the different assays is shown in **Supplementary Table 1**.

### Serology

Anti-SARS-CoV-2 IgG was measured by quantitative anti-SARS-CoV-2 QuantiVac enzyme-linked immunoassay (ELISA) (IgG) based on the SARS-CoV-2 S1 spike protein domain/receptor-binding domain (EI 2606-9601-10G; Euroimmun, Lübeck, Germany) and in accordance with the manufacturer’s instructions (dilution up to 1 : 4,000). In addition, Euroimmun provided us with a quantitative ELISA to assess anti-SARS-CoV-2 S1 Omicron IgG (EI 2606-9601-30G). Anti-S1 concentrations (Relative Units (RU)/mL) were extrapolated from calibration curves, with values below 11 RU/mL defined as negative. Values from the SARS-CoV-2 QuantiVac (RU/mL) were multiplied by 3.2 in order to convert them to binding antibody units per mL (BAU/mL). Anti-SARS-CoV-2 nucleocapsid (NCP) IgG measurements were performed in accordance with the manufacturer’s instructions (Euroimmun, Lübeck, Germany). Aesku.Reader (Aesku.Group, Wendelsheim, Germany), and Gen5 version 2.01 software was used for the analysis.

### Surrogate virus neutralization assay for SARS-CoV-2 variants

To quantify neutralizing antibodies against the Wuhan spike, the B.1.1.7 spike (Alpha), the B.1.351 spike (Beta), the P.1 spike (B.1.1.28.1; Gamma), the B.1.617.2 spike (Delta), and the B.1.1.529 spike (Omicron sublineages BA.1, BA.2, and BA.5) variants of SARS-CoV-2-S in plasma, we modified our recently established surrogate virus neutralization test (sVNT) (3, 7). In this assay, the soluble receptor for SARS-CoV-2, ACE2, is bound to 96-well-plates to which different purified tagged receptor-binding domains (RBDs) of the spike protein of SARS-CoV-2 can bind once added to the assay. Binding is further revealed by an anti-tag peroxidase-labeled antibody and colorimetric quantification. Preincubation of the spike protein with the serum or plasma of convalescent patients or vaccinees prevents subsequent binding to ACE2 to varying degrees, depending on the quantity of neutralizing antibodies present. In detail, MaxiSorp 96F plates (Nunc) were coated with recombinant soluble hACE2-Fc (IgG1) protein at 300 ng per well in 50 μL of coating buffer (30 mM Na_2_CO_3_, 70 mM NaHCO_3_, pH 9.6) at 4°C overnight. After blocking with hACE2-Fc (IgG1), plates were washed with phosphate-buffered saline–0.05% Tween-20 (PBST) and blocked with BD OptEIA Assay Diluent for 1.5 h at 37°C. Meanwhile, plasma samples were serially diluted three times starting at 1 : 6.7 or 1 : 20 and then preincubated for 1 h at 37°C with 1.5 ng of recombinant SARS-CoV-2 spike RBDs from either the Wuhan strain (Trenzyme), the B.1.1.7 variant (N501Y; Alpha), the B.1.351 variant (K417N, E484K, N501Y; Beta), the P.1 variant (K417T, E484K, N501Y; Gamma), the B.1.617.2 variant (L452R, T478K), the B.1.1.529 BA.1 variant (G339D, S371L, S373P, S375F, K417N, N440K, G446S, S477N, T478K, E484A, Q493R, G496S, Q498R, N501Y, Y505H), or the B.1.1.529 BA.2 variant (G339D, S371F, S373P, S375F, T376A, D405N, R408S, K417N, N440K, S477N, T478K, E484A, Q493R, Q498R, N501Y, Y505H; (all spike RBD from variants were obtained from Sino Biological), all with a C-terminal His-Tag. BD OptEIA Assay Diluent was used for preparing the plasma sample and RBD dilutions. After preincubation with SARS-CoV-2 spike RBDs, plasma samples were placed on hACE2-coated MaxiSorp ELISA plates and incubated for 1 h at 37°C. SARS-CoV-2 spike RBDs preincubated with buffer only served as negative controls for inhibition. Plates were washed three times with PBST and incubated with a horseradish peroxidase (HRP)-conjugated anti-His-tag antibody (clone HIS 3D5, provided by Helmholtz Zentrum München) for 1 h at 37°C. Unbound antibody was removed by six washes with PBST. A colorimetric signal was developed on the enzymatic reaction of HRP with the chromogenic substrate 3,3′,5,5′-tetramethylbenzidine (BD OptEIA TMB Substrate Reagent Set). An equal volume of 0.2 M H_2_SO_4_ was added to stop the reaction, and the absorbance readings at 450 and 570 nm were measured using a SpectraMax iD3 microplate reader (Molecular Devices) using SoftMAX Pro v7.03 software. For each well, the inhibition percentage was calculated from optical density (OD) values after subtraction of background values using the following equation:


Inhibition(%) = (1−sample OD value/average SARS−CoV−2 S RBD OD value) × 100.

Neutralizing sVNT titers were determined as the dilution with binding reduction  >  mean  +  2 SDs of values from a plasma pool consisting of three pre-pandemic plasma samples.

### SARS-CoV-2 protein peptide pools

We ordered 15 amino-acid-(aa)-long and 10-aa-overlapping peptide pools spanning the whole length of the SARS-CoV-2 membrane (-M) (total 43 peptides), nucleocapsid (-N) (total 82 peptides), or envelope (-E) protein (a total of 12 peptides; peptide no. 4 could not be synthesized) from GenScript. All lyophilized peptides were synthesized at greater than 95% purity and reconstituted at a stock concentration of 50 mg/mL in dimethylsulfoxide (DMSO) (Sigma-Aldrich), except for two SARS-CoV2-M peptides (nos. 15 and 16), one SARS-CoV2-N peptide (no. 61), and all 12 SARS-CoV2-E peptides, which were dissolved at 25 mg/mL due to solubility issues. All peptides in DMSO stocks were stored at −80°C until use.

### T-cell restimulation assay

After isolation by Ficoll gradient centrifugation, PBMCs were resuspended in complete RPMI medium [RPMI 1640 (Gibco)] supplemented with 1 mM sodium pyruvate, 50 µM β-mercaptoethanol, 10% fetal bovine serum (FBS) (GE Healthcare Life Sciences), and 1% streptomycin–penicillin (all from Gibco) at a concentration of 20 × 10^6^ cells per mL. For stimulation purposes, cells were diluted with peptide pools of equal volume containing a mixture of M-, N-, and E-proteins or using PepTivator^®^ Peptide Pools for SARS-CoV-2 Prot_S B.1.1.529/BA.1 (Miltenyi; no. 130-129-928) or SARS-CoV-2 Prot_S B.1.1.529/BA.1 wild-type (WT) control (Miltenyi; no. 130-129-927). All peptide pools were prepared in complete RPMI with a final concentration of 10 µg/mL brefeldin A (Sigma-Aldrich). The concentration of each peptide in the final PepTivator® Peptide Pools mixture was 1 µg (approximately 0.6 nmol)/mL. The other peptides were present at a concentration of 2 µg (approximately 1.2 nmol)/mL, with the exception of SARS-CoV2-N peptide 61 and SARS-CoV2-M peptides 15 and 16, which were used at a final concentration of 1 µg/mL due to solubility issues. PBMCs were stimulated with DMSO as a negative control for the M-, N-, and E-peptide pool. As an internal positive control, in each experiment, cells were stimulated with ionomycin (Invitrogen) and phorbol 12-myristate 13-acetate (Calbiochem) at a final concentration of 1,500 ng/mL and 50 ng/mL, respectively. For the stimulation, cells were incubated for 12–16 h (at 37°C, in 5% CO_2_). After harvesting, cells were resuspended in MACS buffer (PBS supplemented with 3% FBS and 2 mM EDTA) containing 10% mouse serum and incubated at 4°C for 15 min to avoid non-specific antibody binding. Next, without washing, the previously described antibody mix (7) was added and cells were stained for 20 min at room temperature in the dark. Cells were then washed before they were fixed and permeabilized (no. 554714; BD Biosciences) in accordance with the manufacturer’s protocol. Samples were stained, for the cytokines of interest, intracellularly for 45 min in the dark and at room temperature using the antibodies described previously (7). After washing off excess antibody, samples were analyzed on a five-laser Cytek Aurora spectral flow cytometer (Cytek) (355 nm, 405 nm, 488 nm, 561 nm, and 640 nm). All flow cytometry data were acquired using SpectroFlo version 2.2.0 (Cytek) and analyzed with FCS Express 7 (Denovo). See also our previous work (3, 7).

### Flow cytometric detection and analysis of SARS-CoV-2-specific B cells

SARS-CoV-2-spike-specific B cells were detected using a recombinant, biotinylated SARS-CoV-2 spike (Acro Biosystems: Wuhan—SPN-C82E9; and Omicron—SPN-C82Ee). Recombinant spike Wuhan and Omicron proteins were tetramerized with fluorescently labeled streptavidin (streptavidin, allophycocyanin, cross-linked, catalog no. S868, and streptavidin, R-phycoerythrin conjugate, catalog no. S21388, respectively; ThermoFisher) following the suggested protocol for tetramerization from the National Institutes of Health. Small amounts of streptavidin were added to the solution containing the recombinant proteins every 10 min for a total of 10 times. Volumes were defined taking into account the size of the recombinant proteins and concentration of streptavidin in order to achieve “equal molarity”. Using this protocol, we added excess streptavidin to the solution, but we expected maximal tetramer formation.

PBMCs were thawed, counted, and rested at a concentration of 2 × 10^6^ cells/mL for 6–7 h (at 37°C, in 5% CO_2_) in complete RPMI (as described before). After resting, cells were resuspended in MACS buffer (PBS, 5 mM EDTA, and 3% FBS) and stained for 20 min at room temperature with an antibody mix containing the antibodies listed in **Supplementary Table 2** together with tetramerized recombinant proteins (as described before) against the Wuhan spike and Omicron (B.1.1.529) spike. After one wash, samples were acquired on a spectral flow cytometer and data were analyzed as described below. See also our previous work (3, 7).

### Quantification of IFN-γ release

Using the manufacturer’s selected parts of the SARS-CoV-2 S1 domain of the spike protein (based on the Wuhan strain), 0.5 mL of whole blood was stimulated for a period of 20–24 h [ET 2606-3003, SARS-CoV-2 Interferon Gamma Release Assay (IGRA) (Euroimmun, Lübeck, Germany)]. An additional tube coated with selected parts of the SARS-CoV-2 S1 domain of the Omicron spike protein was used for stimulation (Euroimmun, Lübeck, Germany) spike. We carried out negative and positive controls in accordance with the manufacturer’s instruction and measured IFN-γ using an ELISA (EQ 6841–9601, Euroimmun, Lübeck, Germany). For analysis, we used an Aesku.Reader (Aesku.Group, Wendelsheim, Germany) and Gen5 version 2.01 software.

### Statistics

All statistical analyses were performed using GraphPad Prism version 8.4 or 9.0 (GraphPad Software Inc., CA, USA). A paired *t*-test (within groups) or two-way ANOVA followed by Sidak’s multiple comparison test (between groups) was used for comparing levels of spike-specific IgG, and for comparison of IFN-γ concentration in serum (IGRA). For sVNT titers, we used a paired two-sided *t*-test (within groups). One-way ANOVA followed by a Kruskal–Wallis multiple comparison test was used for the comparison of frequencies of spike-specific IgD^−^ B cells. Percentages of cytokine-secreting T cells were log-transformed prior to comparison. Differences were considered significant at a *p*-value < 0.05.

### Ethics committee approval

The CoCo Study and the analysis conducted for this article were approved by the Internal Review Board of Hannover Medical School (institutional review board no. 8973_BO-K_2020, amendment December 2020). All study participants gave written informed consent and received no compensation.

## Results

We have previously described, in a cohort of healthcare professionals, the longitudinal immune response after one, two, and three immunizations, applying a ChAd/ChAd/BNT, ChAd/BNT/BNT, or BNT/BNT/BNT immunization regimen (7). In the spring of 2022 several study participants reported PCR-confirmed breakthrough infections. As the ChAd/BNT/BNT group was the largest cohort in our study, we decided to focus on and analyze the immune response in individuals in that cohort who experienced a breakthrough infection (Omicron breakthrough infection group) and to compare it with the immune response in age- and sex-matched control subjects without reported infections from the same cohort (triple vaccination group; **Figure 1A**). Seven of the eight individuals with PCR-confirmed COVID-19 seroconverted and developed anti-NCP serum IgG; the remaining individual did not seroconvert despite developing clinical symptoms and presenting a PCR-confirmed SARS-CoV-2 infection (**Supplementary Figure 1A**). Of interest, 3 out of 18 control individuals also seroconverted without having had any clinical symptoms, and were thus assigned to the Omicron breakthrough infection group. Another individual from the control group was later assigned to the Omicron breakthrough infection group because exceptionally high anti-S1 antibodies and IGRA values were found during the analysis. This person did not show anti-NCP IgG seroconversion, but reported a mild pharyngitis. In addition, the immune response of 15 non-immunized individuals infected during late winter/early spring 2022 was also studied 16 to 91 days post confirmed infection. The latter were presumably infected with the Omicron sublineage BA.1 or BA.2, as those were the dominant variants in Germany at the time of infection. This group is referred to as the non-vaccinated, Omicron-infected group (Omicron infection group). In addition, sera and PBMCs from non-vaccinated individuals with mild COVID-19 who were infected at the beginning of the pandemic with the Wuhan strain (Wuhan infection group) were also analyzed in some assays.

In the triple vaccination group, we observed a significant (85.9%) overall reduction in anti-Wuhan-S1-binding antibodies when comparing the titers obtained 14 days after the third vaccination with those obtained 4 months after the third vaccination, resulting in a median of 696 BAU/mL (range 100–1,929 BAU/mL). In contrast, anti-Wuhan-S1 IgG values decreased considerably less in individuals with an Omicron breakthrough infection. Three individuals presented even higher titers after the infection than at 14 days after their third vaccination. Of interest, anti-spike IgG levels in the Omicron infection group were significantly lower (median 28 BAU/mL; range 7–6,153 BAU/mL) and were within the range of those found in the Wuhan-Hu-1 infection group (median 74 BAU/mL; range 0–8,064 BAU/mL) (**Figure 1B**). IGRAs after whole-blood restimulation with Wuhan spike peptides revealed a similar picture: a significant overall decrease of 73.3% was observed in the triple vaccination group within the 3.5-month follow-up period, whereas levels did not change significantly in the Omicron breakthrough infection group in the same timeframe. In contrast to the relatively weak antibody response found in individuals in the Omicron infection group, IFN-γ release values were within the range observed in the triple-vaccinated group 4 months after the last vaccination (**Figure 1C**). Similar results were observed when performing Omicron-based anti-spike IgG ELISA and Omicron-based IGRAs. Omicron breakthrough infections resulted in higher anti-Omicron spike IgG levels than those seen in the triple vaccination group at 4 months’ follow-up. Non-vaccinated individuals after Omicron or Wuhan infection again presented very low levels of Omicron-specific IgG (**Supplementary Figure 1B**). Furthermore, individuals with Omicron breakthrough infections were associated with the strongest IGRA responses after restimulation with Omicron spike protein-derived peptides (**Supplementary Figure 1C**). Notably, neither age nor sex had any significant impact on anti-S1 IgG and IGRA values among the groups analyzed (**Supplementary Figures 2A–H**). In summary, waning anti-spike IgG levels were restored in triple-vaccinated individuals after Omicron breakthrough infection.

**Figure 2:**
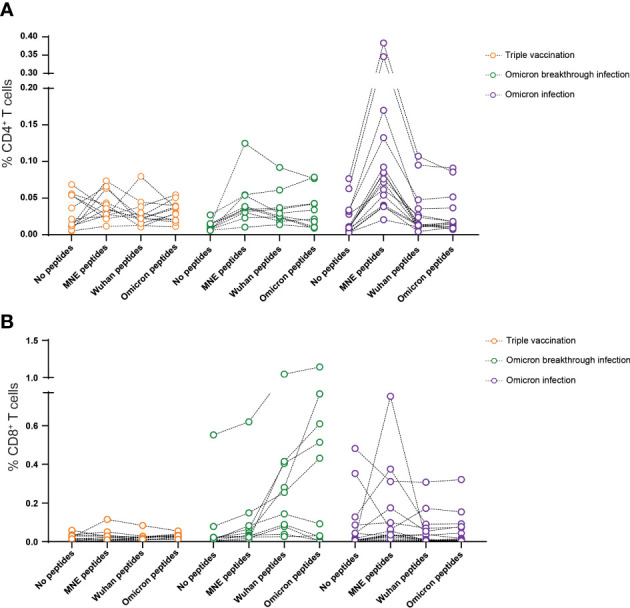
Omicron breakthrough infection in ChAd/BNT/BNT vaccinees leads to moderate increased T-cell responsiveness to SARS-CoV-2. Omicron breakthrough infection in vaccinees induces a negligible cytokine secretion by T cells in response to peptides from the M (membrane), N (nucleocapsid), and E (envelope) of the virus **(A)**, and a small increase in cytokine-secreting CD8 T cells in response to peptides from the Omicron BA.1 variant spike protein **(B)**. We calculated the total number of cytokine-secreting cells as the sum of IFN-γ^+^TNF-α−, IFN-γ^+^TNF-α^+^, and IFN-γ^−^TNF-α^+^ cells. Data are representative of five independent experiments: *n *= 12 for triple-vaccinated non-infected participants, *n*= 10 for triple-vaccinated participants with Omicron breakthrough infection, and *n *= 15 for non-vaccinated participants with Omicron infection. ChAd, AstraZeneca’s Vaxzevria vaccine; BNT, BionTech/Pfizer’s Comirnaty vaccine.

SARS-CoV-2-specific T-cell activity was addressed by restimulating freshly isolated PBMCs with peptide pools derived from the M, N, or E protein or a peptide pool from the spike protein of the Wuhan variant (Wuhan-S) or Omicron variant (Omicron BA.1-S) of SARS-CoV-2. After 12–16 h, the percentage of CD4^+^ or CD8^+^ T cells producing IFN-γ and/or TNF-α was determined. As shown previously, 4 months after the last vaccination, the frequency of cytokine-producing CD4^+^ or CD8^+^ T cells is close to undetectable with this experimental approach (**Figures 2A, B**). In contrast, in the majority of participants from the breakthrough infection group, MNE- and/or S-specific CD4^+^ and CD8^+^ T cells could readily be identified (**Figures 2A, B**). Interestingly, individuals of the Omicron infection group showed high frequencies of CD4^+^ T cells specific for MNE-peptides. Of note, comparing CD4^+^ and CD8^+^ T-cell response to restimulation did not reveal significant differences between the peptide pools derived from the Wuhan spike protein and those derived from the Omicron BA.1 spike protein (**Figure 2B**).

**Figure 3:**
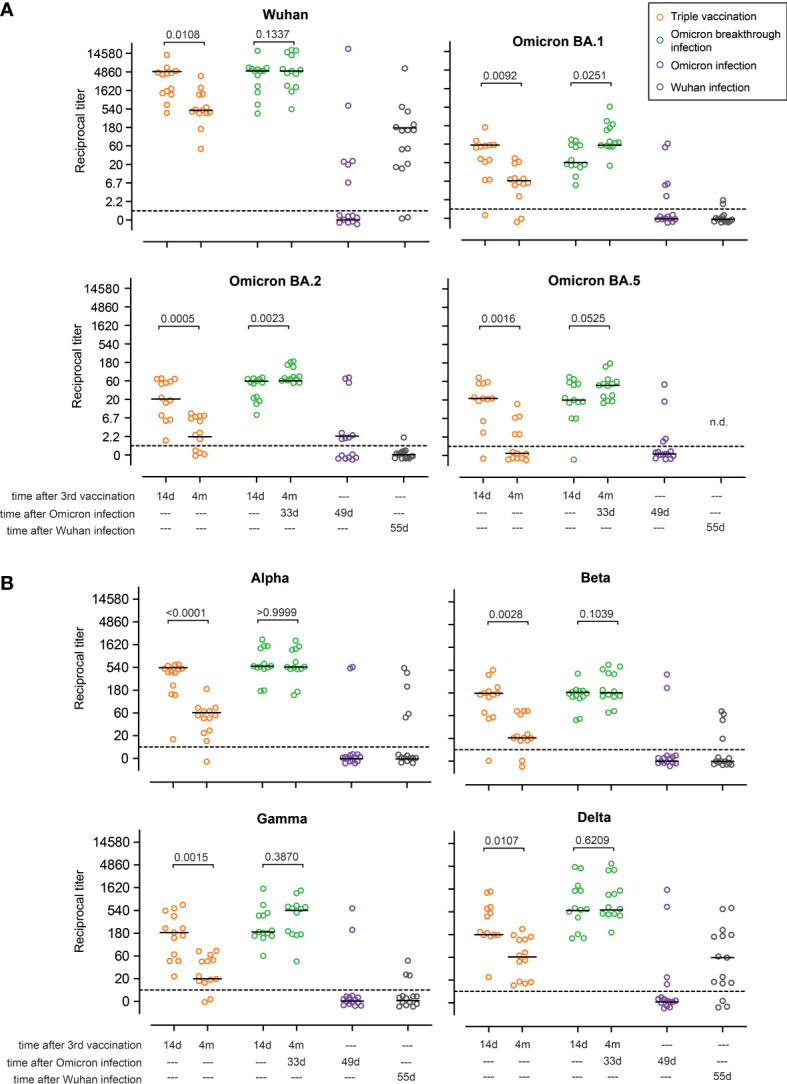
Omicron breakthrough infection in ChAd/BNT/BNT vaccinees restores neutralizing antibodies. Omicron infection in ChAd/BNT/BNT vaccinees results in a strong immune boost, leading to the induction of neutralizing antibodies against **(A)** Wuhan and B.1.1.529 (Omicron) BA.1, BA.2, and BA.5 sublineages, and also **(B)** B.1.1.7 (Alpha), B.1.351 (Beta), P.1 (B.1.1.28.1; Gamma), and B.1.617.2 (Delta) SARS-CoV-2-S variants measured using the surrogate virus neutralization test (sVNT). For better visualization of identical titer values, data were randomly and proportionally adjusted closely around the precise titer results. The dotted line represents the lower limit of detection. The black lines represent the group median. A paired two-sided *t*-test was performed within groups. ChAd, AstraZeneca’s Vaxzevria vaccine; BNT, BionTech/Pfizer’s Comirnaty vaccine.

We next tested serum antibodies for their neutralizing capacity, applying surrogate virus neutralization tests (sVNTs) as described before. This test is an ELISA-based assay in which plates are coated with ACE2 and sera are tested for their ability to block the binding of soluble RBDs encoded by various SARS-CoV-2 variants. Between 14 days and 4 months post vaccination, sera from individuals in the triple vaccination group exhibited a ninefold reduction (two titer steps) in neutralizing capacity against the Wuhan, Omicron BA.1, Omicron BA.2, Alpha, Beta, Gamma, and Delta variants, and decreased below the limit of detection for Omicron BA.5 (**Figures 3A, B**). In contrast, no titer decline was observed in the Omicron breakthrough infection group for any of the variants tested. Interestingly, a threefold increase (one titer step) was observed for the Gamma and Omicron BA.1 variants (**Figures 3A, B**). We also tested sera from individuals in the Omicron infection group and compared them with sera from individuals in the Wuhan infection group. The latter samples were collected from patients with mild disease following infection with the Wuhan strain during the first months of the pandemic. Interestingly, less than 50% of the samples from the Omicron infection group contained measurable neutralizing capacity against Wuhan, Omicron BA.1, and Omicron BA.2. In fact, only 2/15 participants had measurable titers of neutralizing capacity against Alpha, Beta, and Gamma, and only 4/15 participants had measurable titers of neutralizing capacity against Delta and Omicron BA.5. On the other hand, within the Wuhan infection group, 13/15 individuals had measurable titers of neutralizing capacity against the Wuhan strain, while only one and two participants showed measurable titers of neutralizing capacity against Omicron BA.1 and BA.2, respectively (**Figures 3A, B**). Out of 15 sera analyzed, five had measurable titers of neutralizing capacity against Alpha, four against Beta, and three against Gamma, whereas 12 had titers against Delta. Altogether, these data show that Omicron breakthrough infection in ChAd/BNT/BNT vaccinees resulted in a strong boost of the humoral immune response, with neutralizing capability against all variants tested. However, when compared with other groups, neutralization titers against Omicron BA.1, BA.2, and BA.5 were low (**Figures 3A, B**). In contrast, approximately only half of the non-vaccinated individuals with Omicron infection developed measurable titers against the Omicron and the Wuhan variants, and even fewer developed neutralizing antibodies against the other variants. Non-vaccinated individuals from the Wuhan infection group developed higher neutralization titers against Wuhan and Delta than non-vaccinated individuals after Omicron infection, but developed hardly any neutralizing antibodies against Omicron variants.

**Figure 4:**
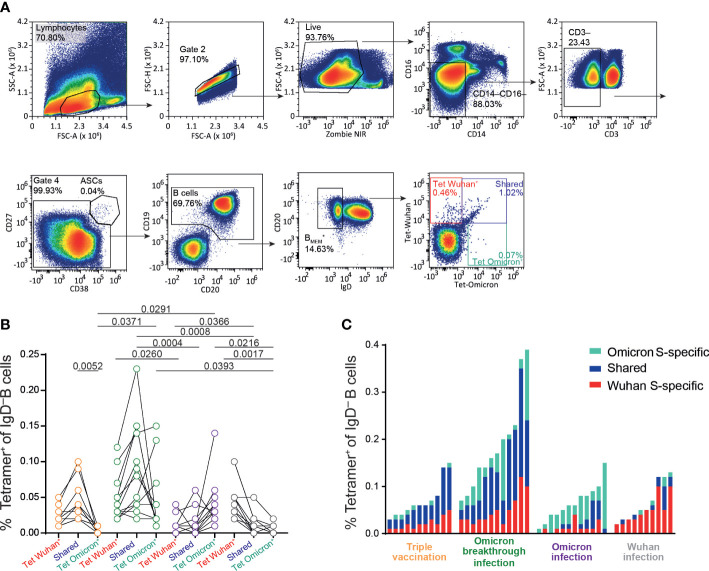
Increase in B.1.1.529-spike specific memory B cells after infection in vaccinated individuals. Frozen peripheral blood mononuclear cells (PBMCs) samples from Omicron-convalescent and “naive” individuals triple vaccinated with the Chad/BNT/BNT regime, and Omicron-convalescent and Wuhan-convalescent non-vaccinated individuals were thawed, rested at 37°C for 6 h and stained for extracellular markers together with fluorochrome-labeled SARS-CoV-2 S protein tetramer (Wuhan and B.1.1.529 variants). **(A)** Gating strategy for SARS-CoV-2-S (spike)-specific IgD^−^ B-cell populations in PBMCs (sample from a vaccinated non-infected individual). **(B)** Frequency of IgD^−^ B cells binding Wuhan, Omicron, or both (shared) full-length S proteins for vaccinated individuals not infected (orange) and infected (green) with Omicron, and non-vaccinated individuals infected with Omicron (purple) and Wuhan (gray) strains. **(C)** Total frequencies of full-length spike-specific IgD^−^ B cells in non-infected and infected vaccinated individuals, and non-vaccinated individuals infected with Omicron or Wuhan strains (same data presentation as in B). Data are representative of five independent experiments: *n* = 11 for triple-vaccinated non-infected participants, *n* = 12 for vaccinated and Omicron-infected participants, *n* = 12 for non-vaccinated Omicron-infected participants, and *n* = 10 for non-vaccinated Wuhan-infected participants. b, One-way ANOVA followed by a Kruskal–Wallis multiple comparison test. ChAd, AstraZeneca’s Vaxzevria vaccine; BNT, BionTech/Pfizer’s Comirnaty vaccine.

Finally, we characterized the B-cell response in triple vaccinees with or without Omicron breakthrough infection and in individuals of the Omicron or Wuhan infection group. For this, we used tetramerized recombinant spike proteins from the Wuhan and Omicron (BA.1) variants to determine the frequency of memory B cells carrying B-cell receptors binding specifically to either one or both spike proteins (**Figure 4A**). As expected, PBMCs from individuals in the triple vaccination group possessed B cells recognizing selectively either the Wuhan spike (Wuhan-S specific) or Wuhan and Omicron spike proteins (shared), whereas B cells selectively recognizing the Omicron spike protein (Omicron-S specific) were largely absent in this group. On the other hand, in addition to the Wuhan-S-specific and shared populations, individuals with Omicron breakthrough infection had additional B cells that also selectively recognized the Omicron spike protein. In contrast, individuals of the Omicron or Wuhan infection groups in most cases had low frequencies of spike-specific B cells, and the large majority of these cells were specific to the spike protein of the virus variant causing the infection (**Figures 4B, C**). Overall, the proportion of exclusively Omicron-specific to Wuhan/Omicron-specific B cells was higher in non-vaccinated individuals with Omicron infection than in those who had experienced breakthrough infection (**Figure 4C**). Comparison of triple-vaccinated individuals with or without Omicron infection revealed that exposure to the Omicron spike protein primarily leads to the expansion of B cells that recognize the spike protein of both Wuhan and Omicron, whereas B cells selectively binding to the Omicron spike are rare. Given the currently dominating Omicron variants, these data support the broad application of Omicron-adapted COVID-19 vaccines. However, due to the strong Wuhan spike bias in triple-vaccinated individuals, our current data question the suitability of bivalent vaccines that also contain information on the Wuhan spike.

## Discussion

In the present study, we analyzed the immune response in triple-vaccinated individuals with and without Omicron breakthrough infections and compared it with the immune response in non-vaccinated patients with mild COVID-19 symptoms who had been infected with a recent Omicron variant or the original Wuhan virus at the beginning of the pandemic. We show that breakthrough infection re-establishes B- and T-cell responses to levels equal to or above those observed 2 weeks after the third vaccination. In contrast, in triple-vaccinated individuals without breakthrough infection, approximately 90% of the antibody response (anti-S1, sVNT) and 70% of the T-cell response (IGRA) were lost within the 3.5–4 months after vaccination. Of interest, among triple-vaccinated individuals, the magnitude of neither the B-cell response nor the T-cell response measured 14 days after the last vaccination correlated with susceptibility to or protection from infection. This however is not surprising considering the neutralization results reported in the present study and by others (10), demonstrating that anti-S1 antibodies induced by first-generation COVID-19 vaccines—all of which were based on the Wuhan virus or the Wuhan virus spike protein—show little neutralizing activity against all Omicron variants tested. Our data also show that non-vaccinated individuals infected with Omicron (most likely BA.1 or BA.2) developed only very low Omicron-neutralizing titers, particularly against the BA.4 and the BA.5 variants, which encode for an identical spike protein. A similar picture was observed for triple-vaccinated persons without breakthrough infection. The simultaneous application of tetramerized RBDs of the Wuhan strain and the Omicron BA.1 strain in flow cytometry revealed that a considerable proportion of the triple-vaccinated individuals with breakthrough Omicron-infections were able to mount B-cell responses specific to the Omicron spike protein, which is in agreement with the increased neutralization titers against Omicron.

Recent studies (11–14) have observed a limited variant-specific cross-neutralizing immunity after Omicron breakthrough infection. In line with our data, we suggest that Omicron breakthrough infections are less immunogenic than, for example, Delta, thus providing reduced protection against reinfection or infection from future variants. Results reported by Kahn et al. indicate that hybrid immunity formed by vaccination and Omicron BA.1 infection should be protective against various variants (15). In contrast, Omicron BA.1 infection alone offers limited cross-protection, as unvaccinated participants infected with Omicron BA.1 showed low neutralization capacity against Omicron BA.2, other variants of concern, and ancestral viruses. Others provided evidence for immunological imprinting, as most antibodies derived from the memory B cells or plasma cells of Omicron breakthrough cases cross-react with the Wuhan-Hu-1, BA.1, and BA.2 RBDs, whereas Omicron primary infections elicit the production of B cells of narrow specificity (16, 17).

The differences in immunity between unvaccinated individuals infected with Omicron BA.1 and vaccinated individuals with BA.1 breakthrough infection is concerning (18). As a result of waning immunity, unvaccinated individuals post Omicron infection are likely to have poor cross-protection against existing and possibly emerging SARS-CoV-2 variants, despite acquiring some neutralizing immunity to the infecting Omicron BA.1 variant. Interestingly, other findings suggest that exposure to Omicron BA.2, in contrast to the BA.1 spike glycoprotein, triggers significant N-terminal domain-specific recall responses in vaccinated individuals and thereby enhances the neutralization of BA.4/BA.5 sublineages (19). Wang et al. (18) found that a fourth antigenic exposure to Omicron BA.1 infection increased variant-specific plasma antibody and memory B-cell responses. However, the fourth exposure did not increase the overall frequency of memory B cells or their general potency or breadth compared with a third mRNA vaccine dose. In summary, our data and other published data suggest that a variant-specific mRNA vaccine boost will increase plasma-neutralizing activity and memory B cells that are specific to the variant and closely related strains, but may not elicit the production of memory B cells with better general potency or breadth than the Wuhan-Hu-1-based mRNA vaccine.

As Omicron BA.5 is the dominant variant in many regions of the world, as of early autumn 2022, it seems appropriate that a booster immunization with BA.4/5-adapted vaccines should be considered in all triple-vaccinated individuals without breakthrough infection and all infected non-vaccinated patients, in order to reduce the risk of SARS-CoV-2 infections and severe COVID-19 in the following months. It will be interesting to observe how effectively neutralizing B-cell responses after vaccination with BA.5-adapted vaccines develop in individuals who so far have been neither vaccinated nor infected with any of the SARS-CoV-2 variants, or who have not been vaccinated and infected only with Omicron BA.5. Such studies would gain “real-world data” on the potential impact of a phenomenon known as “original antigenic sin”, i.e., on the magnitude of immune responses toward novel epitopes—such as those needed to neutralize the Omicron BA.5 spike—against a background of many conserved epitopes delivered by previous infections and/or vaccinations. Since BA.5-adapted COVID-19 vaccines have been licensed without prior clinical testing, it will also be interesting to follow how well they can prevent infections at a population level, as this population currently exhibits considerable heterogeneity regarding COVID-19 vaccination and/or SARS-CoV-2 infection history, particularly given the bivalent nature of these vaccines.

## Data availability statement

The raw data supporting the conclusions of this article will be made available by the authors, without undue reservation.

## Ethics statement

The studies involving human participants were reviewed and approved by the Internal Review Board of Hannover Medical School (institutional review board no. 8973_BO-K_2020, amendment December 2020). The patients/participants provided their written informed consent to participate in this study.

## Author contributions 

Study design: GMNB and RF. Data collection: JB-M, SH, GMR, AC, LH, IO, MK, MS, CR, MF, IR, AS, JR, AJ, SW, GB, RL, and BB. Data analysis: GMNB, JB-M, SH, AC, IO, GMR, MH, and MS. Data interpretation: RF and GMNB. Writing: GMNB and RF, with comments from all authors. All authors contributed to the article and approved the submitted version.
