# Supplementary material for: Omicron infection-associated T- and B-cell immunity in antigen-naive and triple-COVID-19-vaccinated individuals
Source: Front Immunol. 2023 May 5;14:1166589. doi: 10.3389/fimmu.2023.1166589 (PMC10196199; doi:10.3389/fimmu.2023.1166589)
Supplement: Supplementary file 1 [file DataSheet_1.docx]

**Suppl. Fig. 1**

**
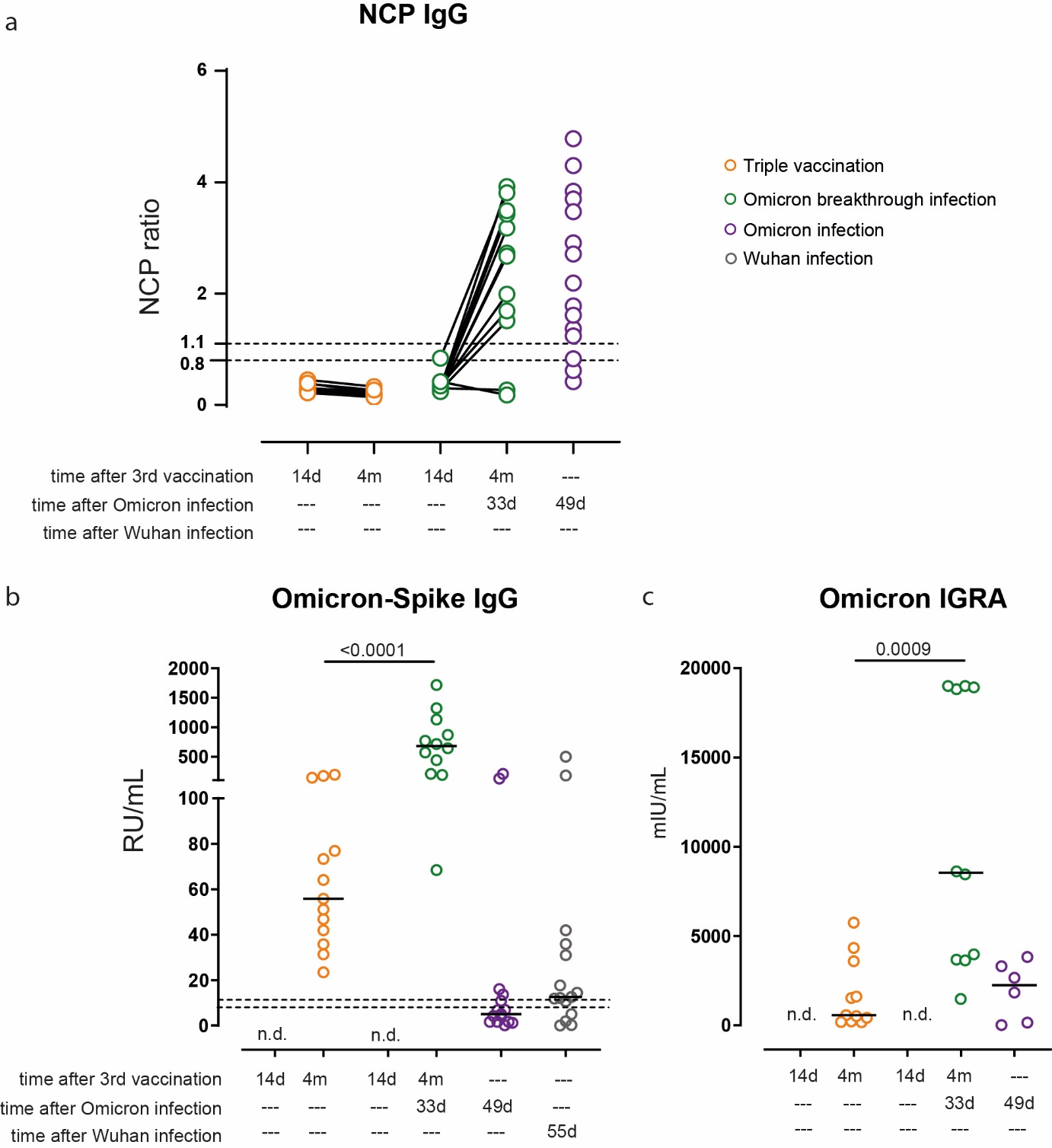
**

**Suppl. Fig. 1 | anti-NCP IgG titers and Omicron-Spike specific immune response after Omicron and Wuhan infection.** a, Antibody levels were determined in a semi-quantitative Anti-SARS-CoV-2-NCP-IgG ELISA. The NCP ratio corresponds to the absorbance of the sample divided through the absorbance of the calibrator. Ratio < 0.8: seronegative; Ratio ≥ 0.8 to < 1.1: borderline; Ratio ≥ 1.1: seropositive. b, anti-S1 Omicron IgG levels measured by ELISA in the plasma. Cut-offs indicated by the dotted lines: < 8 RU/mL: seronegative; ≥ 8 RU/mL to < 11 RU/mL: borderline; ≥ 11 RU/mL: seropositive. c, IFN-γ concentration in full blood supernatants after stimulation with SARS-CoV-2 S1 domain of the Omicron variant for 20–24 h measured by IGRA. n.d., not determined. b, c: ordinary one-way ANOVA followed by Tukey’s multiple comparison test; lines represent group median.

**Suppl. Fig. 2**


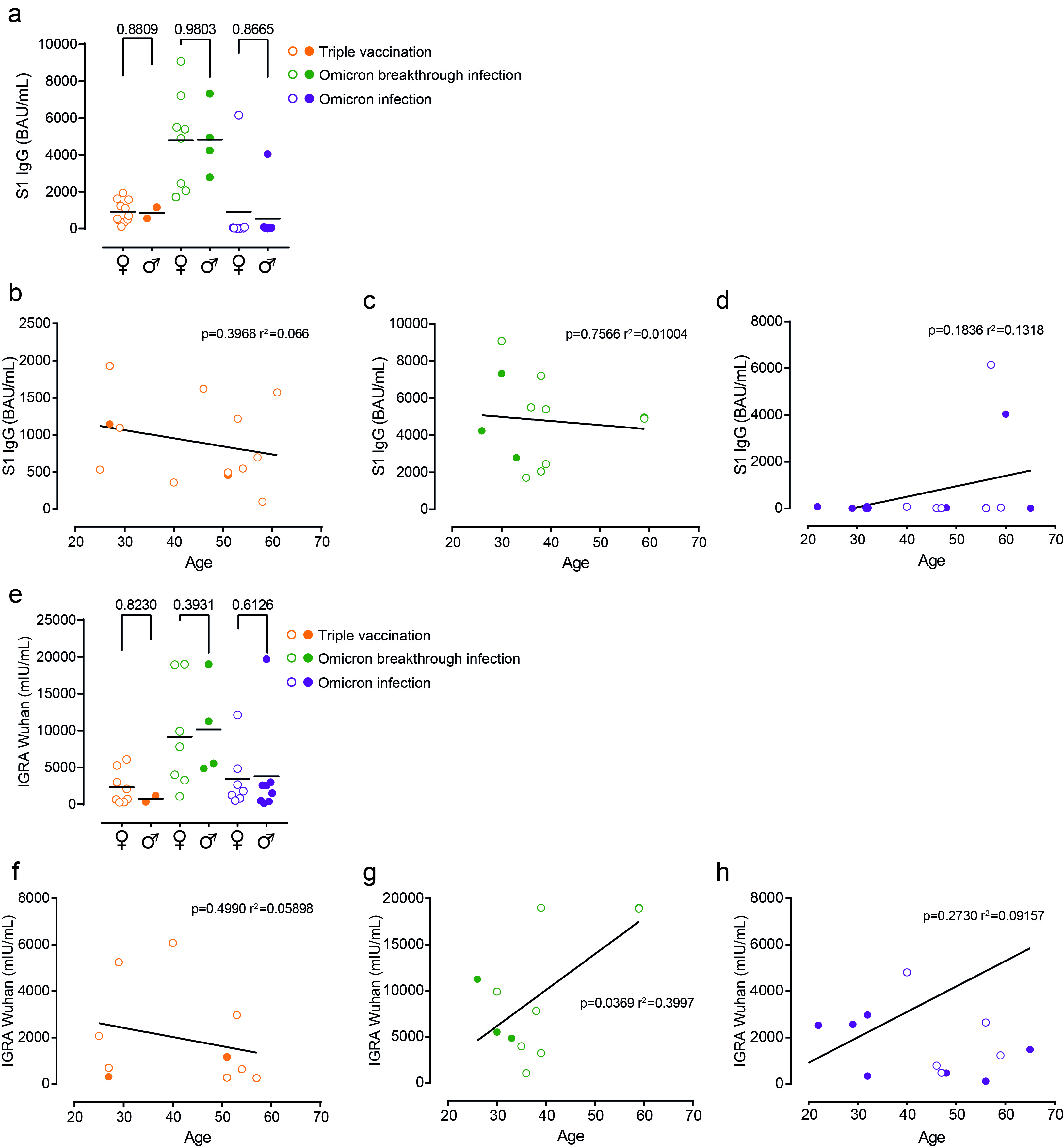


**Suppl. Fig. 2 | Correlation between age and sex with S1 IgG titers and Wuhan-Spike specific immune response after Omicron infection in vaccinated and non-vaccinated individuals and non-infected vaccinated individuals. a**, Distribution of anti-S1 Wuhan IgG levels measured by ELISA in the plasma among the study groups depending on the sex of the participants. **b-d**, Correlation between age and anti-S1 Wuhan IgG levels for Triple vaccinated (**b**), Omicron breakthrough infection (**c**) and Omicron infection (**d**) groups. Filled dots represent male participants and open dots represent female participants. **e**, Distribution of the IFN-γ concentration in full blood supernatants after stimulation with SARS-CoV-2 S1 domain of the Wuhan strain, among the three study groups depending on the sex of the participants. **f-h**, Correlation between age and IFN-γ concentration in full blood supernatants after stimulation with SARS-CoV-2 S1 domain of the Wuhan strain for Triple vaccinated (**f**), Omicron breakthrough infection (**g**) and Omicron infection (**h**) groups.

**Suppl. table 1** | Sample information and number of biologically independent samples analysed from each group for the study in the assays indicated.

| **Group** | **Triple vaccination (n=13)** | | **Omicron breakthrough infection (n=13)** | | **Omicron infection (n=15)** | **Wuhan infection (n=15)** |
| --- | --- | --- | --- | --- | --- | --- |
| Time after 3rd vaccination | 14 d | 4 mo | 14 d | 4 mo | ----- | ----- |
| Time after Omicron infection | ----- | ----- | ----- | 33 d | 49 d | ----- |
| Time after Wuhan infection | ----- | ----- | ----- | ----- | ----- | 55 d |
|  |  |  |  |  |  |  |
| anti-S1-IgG | 13 | 13 | 13 | 13 | 15 | 15 |
| IGRA | 11 | 10 | 11 | 11 | 15 | n.d. |
| Anti-NCP-IgG | 13 | 13 | 13 | 13 | 15 | 15 |
| Anti-Omicron-S1-IgG | n.d. | 13 | n.d. | 12 | 15 | 15 |
| Omicron IGRA | n.d. | 11 | n.d. | 10 | 6 | n.d. |
| sVNT Wuhan | 13 | 13 | 13 | 13 | 15 | 15 |
| sVNT Alpha | 13 | 13 | 13 | 13 | 15 | 15 |
| sVNT Beta | 13 | 13 | 13 | 13 | 15 | 15 |
| sVNT Gamma | 13 | 13 | 13 | 13 | 15 | 14 |
| sVNT Delta | 13 | 13 | 13 | 13 | 15 | 15 |
| sVNT Omicron BA.1 | 13 | 13 | 13 | 13 | 15 | 15 |
| sVNT Omicron BA.2 | 13 | 13 | 13 | 13 | 15 | 15 |
| sVNT Omicron BA.5 | 12 | 13 | 13 | 13 | 15 | n.d. |
| Spike-specific CD4+ cells | n.d. | 12 | n.d. | 10 | 15 | n.d. |
| Spike-specific CD8+ cells | n.d. | 12 | n.d. | 10 | 15 | n.d. |
| Spike-specific B cells | n.d. | 11 | n.d. | 12 | 12 | 10 |

IGRA, Interferon Gamma Release Assay; NCP, nucleocapsid; sVNT, surrogate virus neutralization test; n.d., not determined

**Suppl. table 2** | List of antibodies used for flow cytometry analysis of B cell populations.

| **Target** | **Conjugate** | **Clone** | **Company** | **Catalog n.** | **Lot n.** |
| --- | --- | --- | --- | --- | --- |
| CD14 | BB700 | MΦP9 | BD Horizon | 566465 | 1055172 |
| CD16 | BUV496 | 3G8 | BD Horizon | 612944 | 94137 |
| CD19 | PE-Cy7 | HIB19 | BioLegend | 982410 | B3177196 |
| CD20 | BV421 | 2H7 | BioLegend | 302330 | B313870 |
| CD21 | BUV661 | 1O48 | BD Optibuild | 750187 | 295286 |
| CD27 | BUV805 | L128 | BD Optibuild | 748704 | 1270028 |
| CD3 | AF532 | UCHT1 | invitrogen | 58-0038-42 | 2288218 |
| CD38 | APC-Fire810 | HIT2 | BioLegend | 303549 | B345560 |
| IgD | BV480 | 1A6-2 | BD Horizon | 566138 | 117442 |
| IgM | BV785 | MHM-88 | BioLegend | 314544 | B344020 |
| SARS-CoV-2 Spike Trimer | Biotinylated | N/A | Acrobiosystems | SPN-C82E9 | BV3808-20CYF1-YW |
| SARS-CoV-2 Spike Trimer (Omicron) | Biotinylated | N/A | Acrobiosystems | SPN-C82Ee | BV5712a-21C7F1-11B |
| viability | Zombie NIR | N/A | BioLegend | 423106 | B323372 |
